# Detecting and Mapping Invasive Species Across Riparian Corridors via Object Detection Approaches in UAV Imagery: An Example of *Impatiens glandulifera*

**DOI:** 10.1002/ece3.71921

**Published:** 2025-08-13

**Authors:** Jack Cook, Benjamin P. Roberts, Frédéric Labrosse, Neal Snooke

**Affiliations:** ^1^ Institute of Biological Environmental and Rural Science Aberystwyth University Aberystwyth UK; ^2^ UK Centre for Ecology and Hydrology Bangor Gwynedd UK; ^3^ Department of Computer Science Aberystwyth University Aberystwyth UK

**Keywords:** computer vision, invasive species management, remote sensing, riparian zones, unmanned aerial vehicles (UAVs)

## Abstract

Riparian zones in the United Kingdom have high species diversity but are prone to anthropogenic changes and alien plant invasions, like *Impatiens glandulifera* . However, identification can be challenging due to poor accessibility or visibility via tree canopies. UAVs provide a means to access previously inaccessible areas and capture imagery of the area. In this study, a method is introduced to identify the flowers of invasive species ( *Impatiens glandulifera* ) and map their locations using a computer vision framework and oblique image capture methods. The process includes thresholding images, image masking, blurring, ellipsoid shape search, noise reduction, and contour extraction. Locations are determined using camera parameters, EXIF data, and the average flower size, then converted into vector format for GIS software. This method is wrapped into a single executable program named the semi‐automatic thresholding tool (SATT). A validation set of 312 UAV images from the River Elwy, North Wales, showed high precision (79%–96%) and mean average precision (mAP) scores of 73%–86%. This demonstrates that the SATT consistently and correctly identifies *Impatiens glandulifera* flowers from UAV imagery, making it effective for identifying hotspots and targeting management techniques along riparian corridors. The tool has been wrapped into a single‐file executable program with a graphical user interface, enabling nonexperts to use the tool without the need of any software installation. Overall, the tool obtains consistent detection levels of abundance/or flower density across the study site. The tool also does not require an extensive amount of training data, and the intuitive design of the software enables nonexperts to utilize the tool and modify parameter values to adapt it to their needs.

## Graphical abstract

Riparian zones in the United Kingdom are diverse but prone to anthropogenic changes and invasive species like *impatiens glandulifera* . This study introduces a method to identify and map these flowers using a computer vision framework and UAV imagery, wrapped into an executable program called the semi‐automatic thresholding tool (SATT). Validation showed high precision and consistent identification, making the SATT effective for targeting management techniques along riparian corridors and accessible for nonexperts.

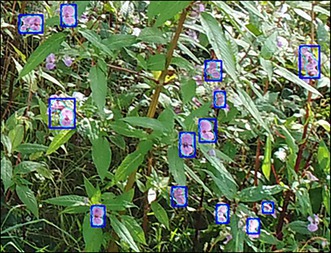

## 1 Introduction

The biological invasion of non‐native plants has caused conservation and economic concerns on a global scale (Cardinale et al. 2018). Within Europe, over 10,822 invasive alien species have been identified (Early et al. 2016). Invasive species are the cause of numerous impacts, including native fauna decline, local flora extinction, as well as hydrological, fire regime, and soil chemistry alterations (Ehrenfeld 2010; Crystal‐Ornelas and Lockwood 2020). These impacts not only threaten biodiversity but also disrupt ecosystem functions and services essential for human well‐being (Ehrenfeld 2010). The resources to counter these invasions are restricted, and eradication schemes can become costly. In the United Kingdom alone, the annual cost associated with invasive non‐native species accounts for £1.7 billion (Williams et al. 2010).

The incidence of invasions by non‐native species has escalated, predominantly driven by the movement of goods and people through global trade and travel, which facilitates the long‐distance dispersal of seeds (Keller et al. 2011). Furthermore, climate change has altered environmental conditions, enabling invasive species to thrive in habitats and locations where survival was previously untenable. Additionally, human disturbances resulting in habitat modification have created more conducive environments for the establishment of invasive species (Didham et al. 2005).

Riparian zones are the interface between terrestrial and aquatic ecosystems (Gurnell et al. 2012; Corenblit et al. 2015). They provide several ecosystem functions relating to biodiversity, water quality, and river flow. These zones are subject to management practices which modify their ecological or hydrological functions, such as logging, grazing, or recreational activities (Michez et al. 2016; Huylenbroeck et al. 2020). Consistent degradation and modification via human disturbance have resulted in these landscape features having heightened invasions from exotic plant species. Flood events within these zones provide an opening for invasive species to establish, whereas also providing propagation opportunities for already established plants (Michez et al. 2016; Zelnik et al. 2020; Wang et al. 2022).

Invasive species management is most cost‐effective if applied as an early intervention in comparison to post‐invasion, long‐term management (Keller et al. 2008). Monitoring provides the possibility for early detection and rapid responses, enabling new infestations to be managed effectively and feasibly (Anderson 2005). Monitoring techniques include predictive modeling, citizen science networks, remote sensing, environmental DNA, and decision‐support systems (Poland et al. 2021).

In the United Kingdom, more than 130 invasive alien species have been monitored along the freshwater network (Gallardo and Aldridge 2020). The most common of these invasive species include *Rhododendron ponticum* (rhododendron), *Fallopia japonica* (japanese knotweed), *Impatiens glandulifera Royle* (himalayan balsam), *Hydrocotyle ranunculoides* (floating pennywort), and *Heracleum mantegazzianum* (giant hogweed) (Cortat et al. 2010; Seeney et al. 2019; Casati et al. 2022). Typically, these invasive species alter the surrounding riparian plant community by displacing native species. They employ various competitive strategies, such as rapid growth, monoculture formation, and wide climate and soil tolerance (Seeney et al. 2019).

Field observations and reporting through paid surveys or citizen‐led data collection dominate the primary data collection for invasive plant species (Crall et al. 2011). However, the data quality and accuracy within these citizen‐led programs can vary, and the programs are often expensive and influenced by observer and sampling bias (Kattenborn et al. 2019; Reaser et al. 2020). Furthermore, in the case of riparian areas, access is often difficult and hazardous for human surveyors, whereas dense canopy coverage can make identification and monitoring of invasive species challenging, which further limits assessment capabilities and therefore restricts the ability to undertake large‐scale surveys at reasonable cost.

To overcome these challenges associated with field monitoring, remote sensing (typically via satellite imagery) has been used as a tool to monitor riparian zones (Kattenborn et al. 2019). This involves imagery being captured from above and analyzed using a host of differing techniques, such as support vector machines for image classification. Typically, studies focusing on extracting features associated with vegetation structure (shade, roughness, height) incorporate LiDAR data, whereas studies on species composition commonly use high‐resolution multispectral images (collected from satellites, planes or UAVs) (Pettorelli et al. 2014; Huylenbroeck et al. 2020). Satellite imagery with pixel resolution smaller than 10 m can provide a means to detect and monitor some invasive plants. However, this technique of distribution mapping via satellite imagery is limited to large species patches or stand sizes, to a single biological community, and not along community boundaries (e.g., riverbanks). This technique also requires the identification of either a unique temporal or spectral feature to differentiate the invasive species from other species and a nonobstructed view, which may be caused by tree canopies (Bradley 2014; Müllerová et al. 2017).

UAV imagery is a flexible approach to collecting imagery at high temporal and spatial resolutions. The approach enables imagery to be captured at optimal times of the year when the invasive species are within key phenological stages that make them easier to identify, for example, flowering (Michez et al. 2016). The flexibility of UAV surveys also enables understory imagery to be collected from an oblique angle, avoiding canopy obstructions which may occur along riparian corridors (Dai et al. 2020).

Pixel‐based image classification when applied to very high resolution imagery obtained from UAVs can be impacted by “salt and pepper” effects or noise from small shadows, which affects the accuracy of the classification (Banham and Katsaggelos 1997; Van der Sande et al. 2003; Azzeh et al. 2018; Hirayama et al. 2019). Multiple image classification studies utilize orthomosaics for classification purposes. However, the quality of these orthomosaics can be impacted by the spatial resolution and the presence of blurring. High spatial resolution can result in extremely detailed images that may be challenging to process, while blurring can degrade the overall quality of the orthomosaics (Lopatin et al. 2019; Saponaro et al. 2021). These issues are often related to the quality of the data acquisition process. It is also possible to mitigate these challenges by reducing the spatial resolution of the final orthomosaic.

An alternative approach is to use object‐based image classification methods, which define a group of pixels (object) as the minimum processing unit for use in image classification techniques (Blaschke et al. 2014; Alvarez‐Taboada et al. 2017; Zhang et al. 2017). Studies using UAVs and object‐oriented approaches for detecting invasive species have already shown promise. These methods are effective in identifying woody invasive species such as *Pinus radiata* , *Ulex europaeus* , *Acacia dealbata* , and *Hakea sericeadection* through image classification based on vegetation indices (Alvarez‐Taboada et al. 2017; Kattenborn et al. 2019).

Therefore, to identify invasive species in these areas using remote sensing methods, easily identifiable features are key to obtaining a good assessment of abundance. These include a unique shape, color, or size of either the flowers, stem, or leaves of the species in comparison to the surrounding vegetation. From the most common invasive species, many of these have unique characteristics often relating to flower color, size, and shape, such as *Rhododendron ponticum* and *Impatiens glandulifera* . The clustering of these flowers also provides an indication of the likely seed dispersal, and in the case of *Impatiens glandulifera* , can highlight hotspot areas of large stands where the plant has established. This is important due to stands collapsing simultaneously during winter conditions and entering the water system, which results in changes amongst environmental mechanics such as riverway blockages as well as increases to water turbidity, nitrogen, and the risk of eutrophication (Tanner et al. 2008; Skálová et al. 2011; Greenwood et al. 2018). In turn, this impacts the abundance of aquatic communities, primarily invertebrates and fish, and further amplifies habitat degradation (Gallardo et al. 2016). Therefore, management efforts focus on trying to remove the *Impatiens glandulifera* when located. However, poor accessibility to the riverbank and obscuration by tree canopies limit the ability to map its location. In this study, single‐image processing is used alongside an object detection method; therefore, limiting any potential impacts from noise associated with single pixel analysis or orthomosaics generation.

Another key challenge is that given it is land managers who are often the ones with the most need for such analyses, many struggle to identify the appropriate remote sensing method for a particular situation, and for it to be sufficiently user‐friendly to be easily adopted. Therefore, open‐access and robust tools are key to enabling effective uptake of remote sensing of vegetation for riparian managers, and these tools must be adaptable so they can be applied to specific sites and circumstances (Huylenbroeck et al. 2020).

The aim of this study is to produce an open‐access and robust tool adaptable to other invasive species with distinct features in their surrounding landscapes. We introduce a method of semi‐automatic species identification via flower recognition, applied to the case example of *Impatiens glandulifera* . This species, with its uniquely shaped and colored flowers, forms large, tall stands along riparian corridors and is highly abundant in the United Kingdom (Varia et al. 2016). The semi‐autonomous method facilitates rapid assessments of objects/flowers detected within UAV imagery, indicating the number of seeds and potential future plant spread. User‐friendly, tunable processing steps allow easy and efficient analysis iterations, with threshold values for hue, saturation, and lightness adjustable via widget sliders, viewable within the software graphics widget. A trigonometric workflow utilizes reference flower sizes and detected flower sizes to georeference identified flowers within oblique images, converting data into point shapefile format for use in Geographic Information Systems (GIS) software. These techniques have been integrated into an easy‐to‐use software named the Semi‐Automatic Thresholding Tool (SATT). To emphasize the SATT, it facilitates users so they do not need to manually review every UAV survey image, rather than replacing human analysis or operating as a standalone system. The SATT can also be adapted to other species by modifying software parameters, such as the thresholding values and kernel shape. This approach is demonstrated on *Impatiens glandulifera* , utilizing a dataset of 312 images from three UAV survey flights along the River Elwy in Wales.

## 2 Materials and Methods

### 2.1 Image Capture Methods

**FIGURE 1:**
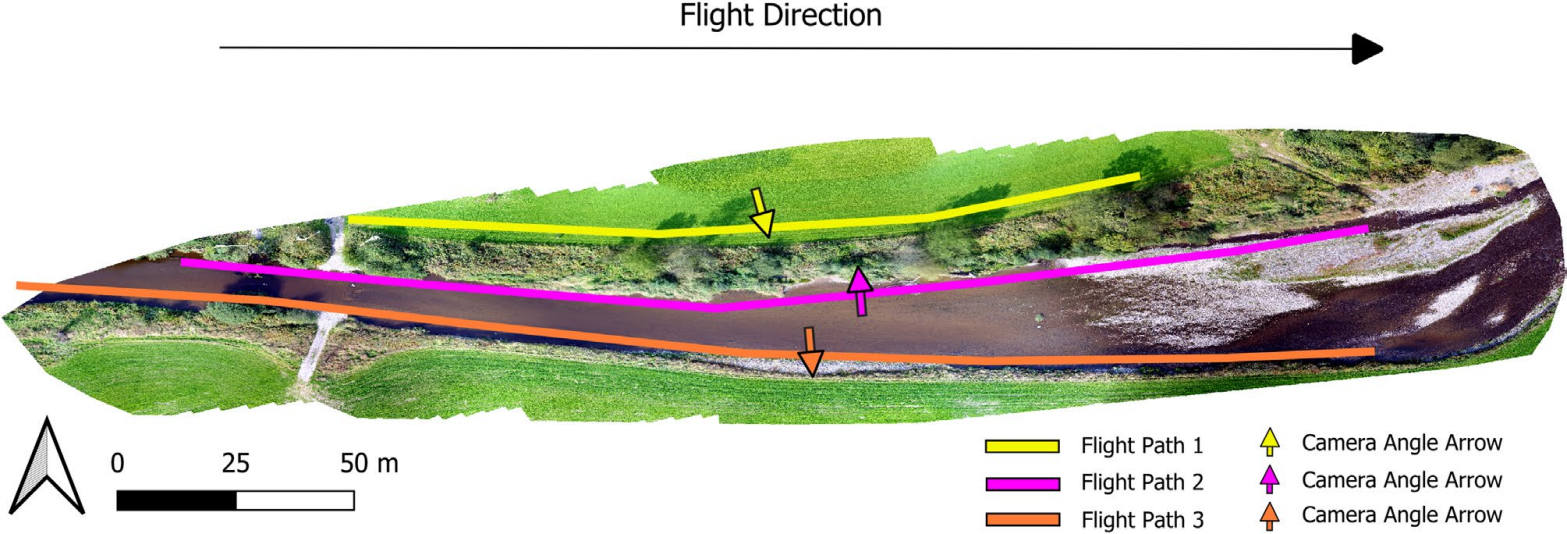
Unmanned Aerial Vehicle (UAV) orthomosaic captured at nadir 30 m above ground level. Colored lines indicate UAV flight paths. Colored arrows indicate camera angle on the UAV.

The imagery, encompassing blue (450 nm ± 16 nm), green (560 nm ± 16 nm), red (650 nm ± 16 nm), red edge (730 nm ± 16 nm), and near‐infrared (840 nm ± 26 nm) bands, was captured using a Phantom 4 pro multirotor UAV along the River Elwy in North Wales (Eastings: 303458.58, Northings: 371039.85, British National Grid—EPSG:27700) on October 25, 2023 between 2:30 and 3:30 PM, as in Figure 1. The UAV was operated manually both in navigation and in image triggering. The UAV was flown at a ~5 m distance from the riverbank using the collision avoidance distance readings on screen as guidance. A target altitude of ~2 m above ground level (AGL) with a ~45‐degree capture angle was attempted throughout to promote consistency in image capture. A total of three oblique surveys were conducted, all facing a single riverbank (2 facing the same bank but from opposite directions).

### 2.2 Survey Timing and Species Considerations

To minimize the overlap with nontarget species and improve the accuracy of identifying Himalayan Balsam, surveys were conducted during October. During this period, Himalayan Balsam is predominantly in bloom, with minimal competition from other flowering plants.

Although there is potential for other flowering species to coexist within the survey areas of Himalayan Balsam, several key factors indicate that their presence may be limited. Himalayan Balsam aggressively covers large areas, creating monotypic stands that outcompete other species (Seeney et al. 2019). This growth habit often leads to reduced biodiversity within these areas (Ehrenfeld 2010). The plant's tall growth creates significant shading, reducing light availability for other plants and preventing them from establishing beneath the Himalayan Balsam canopy (Varia et al. 2016). Additionally, Himalayan Balsam may release chemicals into the soil (allelopathy), inhibiting the growth of other species (Ehrenfeld 2010). Conducting surveys during late August to early October provides a period in which Himalayan Balsam is predominantly in bloom with minimal competition from other flowering plants (Kostrakiewicz‐Gierałt 2015). By this time, most other species will have finished their blooming season, minimizing overlap and enhancing the accuracy of identifying Himalayan Balsam using UAV‐based computer vision. Table 1 provides a summary of comparable species that share habitat with Himalayan Balsam, highlighting their flowering periods, growth forms, and potential habitat overlap.

**TABLE 1:** Flowering periods, growth forms, and habitat overlap of riparian species with Himalayan Balsam ( *Impatiens glandulifera* ).

Riparian species	Flowering period	Growth form	Geography/habitat	Overlap
Himalayan Balsam ( *Impatiens glandulifera* )	July–October	Patch	Wet, disturbed areas (riverbanks, wetlands)	
Common Comfrey ( *Symphytum officinale* )	May–August	Patch	Damp, nutrient‐rich soils (meadows, riverbanks)	Potentially
Rosebay Willowherb ( *Chamaenerion angustifolium* )	June–September	Patch	Disturbed ground (railways, roadsides)	Potentially
Purple Loosestrife ( *Lythrum salicaria* )	July–September	Patch	Wetlands, marshes, water edges	Potentially
Red Campion ( *Silene dioica* )	May–September	Patch/clusters	Damp, shaded environments (woodland edges)	Potentially
Foxglove ( *Digitalis purpurea* )	May–July	Clusters	Damp, shaded areas (woodland clearings)	Potentially

### 2.3 Computer Vision Thresholding Workflow

**FIGURE 2:**
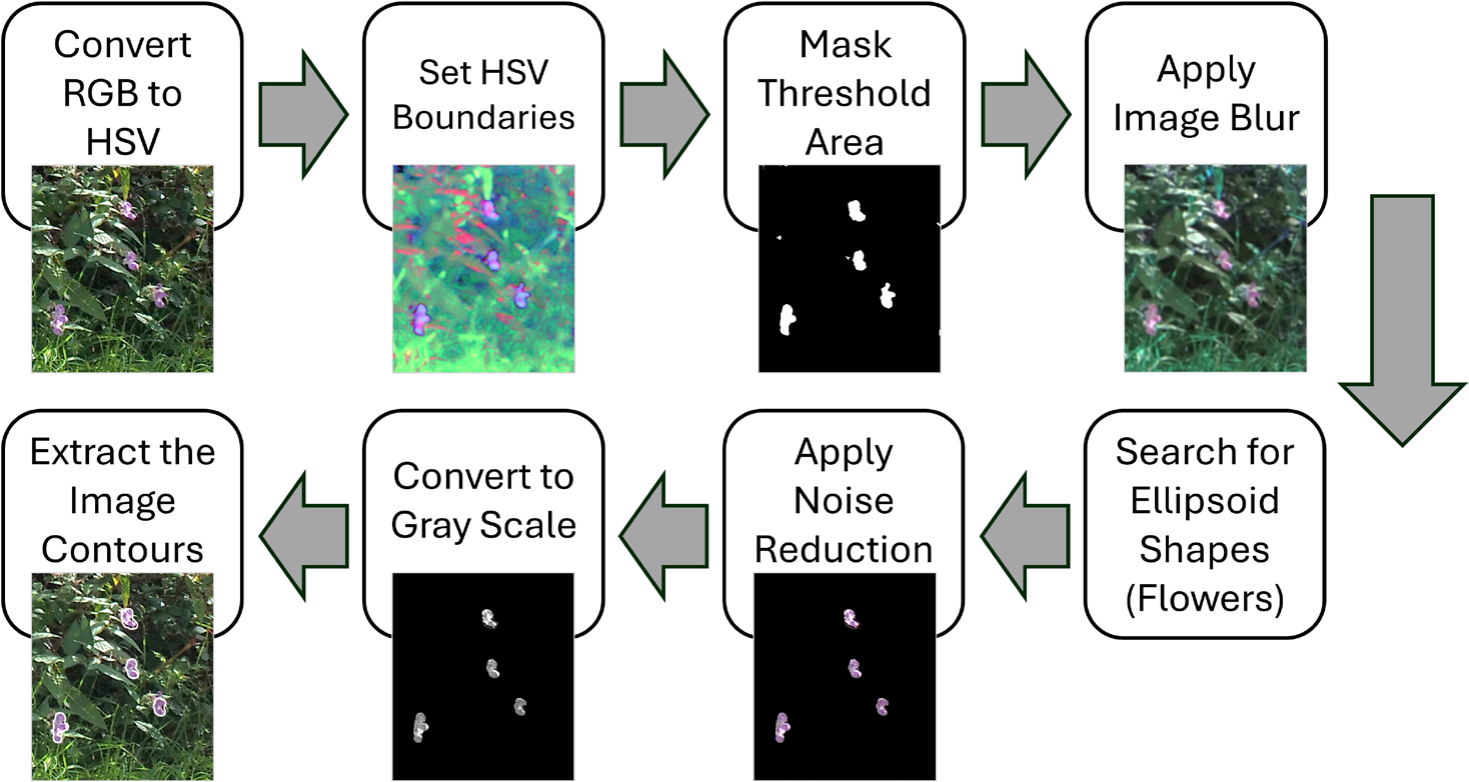
The developed Semi‐Automatic Thresholding workflow.

As the flowers of *Impatiens glandulifera* are the most obvious distinguishable feature for humans of their physiology, investigations focused on methods to extract them from the imagery. Given the distinctive color, initial attempts looked at spectral thresholding. The SATT has been created using Python code. The code utilizes the NumPy (Harris et al. 2020), Pandas (McKinney 2010), GeoPandas (Jordahl 2014), Shapely (Gillies et al. 2007), and OpenCV (Bradski 2000) Python libraries. The ExifTool open‐source software is used to read the embedded metadata of images, also known as the Exchangeable Image File Format (EXIF) (Harvey 2016). The semi‐automatic threshold tool follows the workflow in Figure 2.

The tool utilizes Hue, Saturation, and Value (HSV) color space parameters as well as a kernel search. The choice of HSV color space stems from its ability to separate color information from brightness values, ensuring consistent object color representation despite variations in lighting or reflections. Additionally, HSV enhances robustness in handling shadows, occlusions, and dynamic backgrounds in comparison to the RGB color space. The HSV color space is more correlated with human‐perceived color objects, which links to an important step in the tuning of the SATT software (Hamuda et al. 2017).

User‐defined HSV boundaries via the widget sliders within the software serve as thresholds to mask the image, effectively reducing the search area and minimizing artifacts. The resulting image, reflecting the modifications of boundaries, can be observed via the software's graphics viewer. This functionality enables users to dynamically adjust thresholds in real time until the desired outcomes are achieved. A 15‐pixel Gaussian image blur suppresses high‐frequency noise. Ellipsoid shapes are then searched for within the image using a kernel‐based method as this represents the most similar shape of the *Impatiens glandulifera* flowers in this case study. This method remains robust even when ellipsoids lack perfect definition or contain imperfections (Dassios 2012).

The kernel shape is customizable within the software. In this study, an ellipsoid shape kernel is used as this best represents the shape of *Impatiens glandulifera* , but other examples could include cross‐shaped kernels for flowering species such as *Raphanus raphanistrum* (wild radish) or *Sinapis alba* (white mustard). The kernel size is determined by the input flower (object) reference size, which is a key variable for calculating object distances. This parameter should be adjusted for each plant species under assessment, as flower size varies across species. For rapid assessments, users can apply approximate estimations. Subsequently, a noise reduction filter via opening (erosion followed by dilation) removes isolated pixels or small clusters that could impact overall detection rates. The image is then converted to grayscale to simplify contour detection by reducing it to a single channel, emphasizing object boundaries, and improving processing speed (Tari et al. 1997). Contour extraction is then used to define the edges of objects and locate them within the image, using openCV's findContours function alongside the cv.RETR_TREE contour retrieval mode and the cv.CHAIN_APPROX_SIMPLE contour approximation method.

**FIGURE 3:**
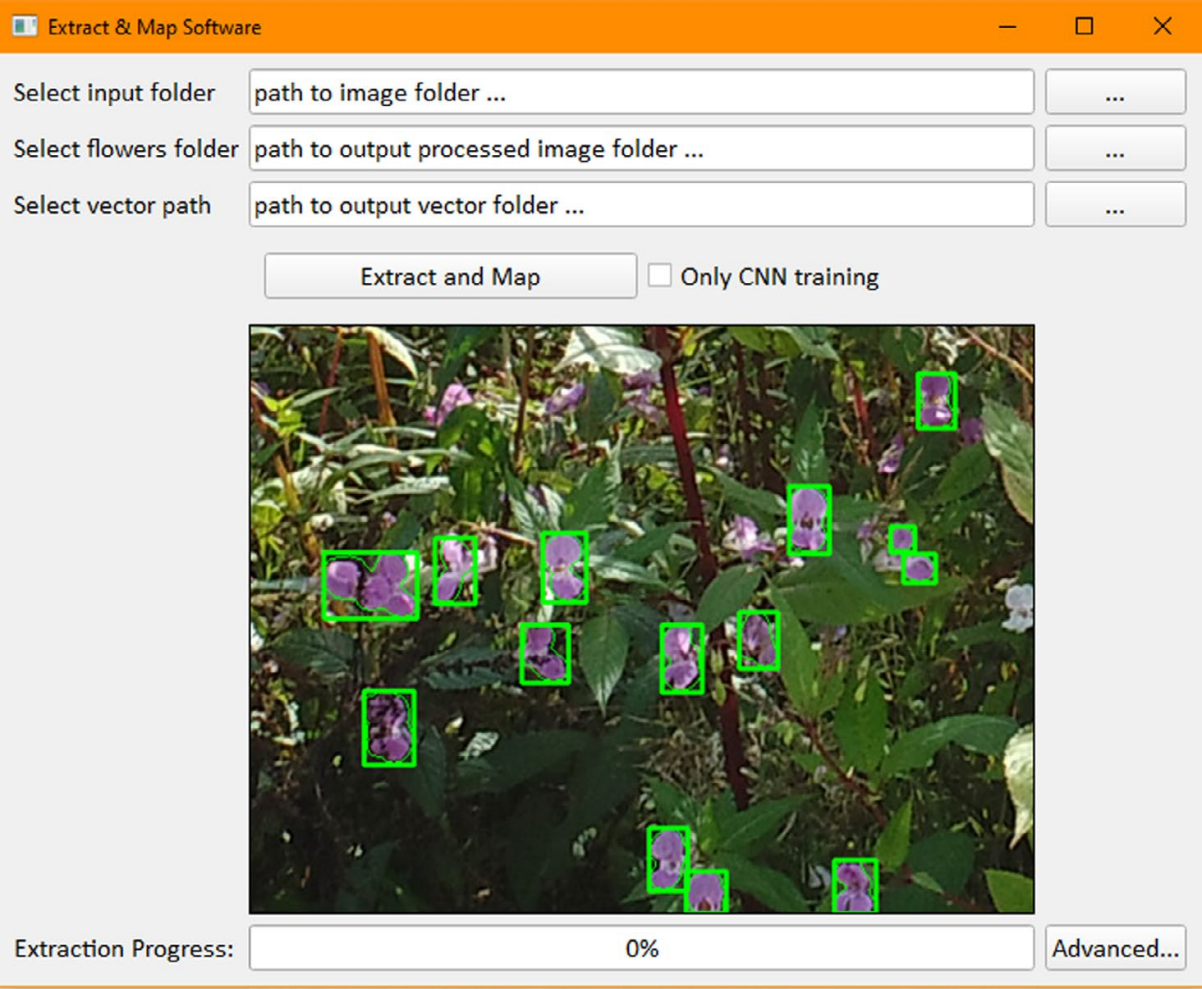
The Graphical User Interface (GUI) for the single‐file executable Semi‐Automatic Thresholding Tool.

**FIGURE 4:**
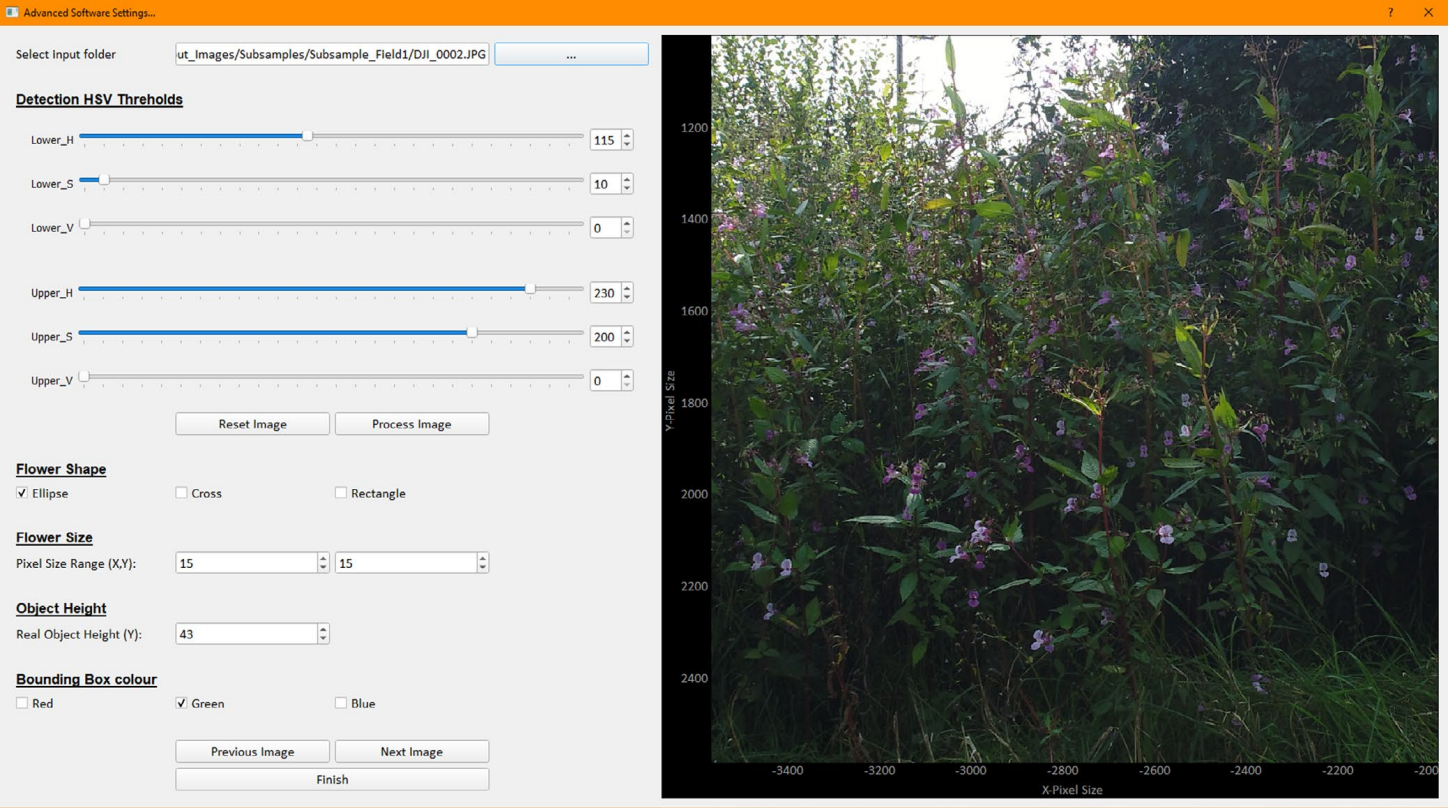
The Advanced popout window used to fine‐tune parameters of the HSV color space thresholds, kernel shape, and minimum object search size (flowers size) measured in pixels, reference object height in mm, and the bounding box color.

The tool has been packaged into a single executable file using the Python PyQt5 library to develop the Graphical User Interface (GUI), as seen in Figure 3, and the python library PyInstaller to compile the software into a single executable file. This enables nonexpert users to utilize the software without needing to install other programs. An “Advanced” set of parameters allows the user to adjust the thresholding parameters, kernel shape, and minimum object size easily and apply this to a subsample of images for initial fine‐tuning of the semi‐automatic workflow in real time to see its effect, as seen in Figure 4. This also allows users to adapt the software to differing plant species.

Once flowers are detected, a bounding box is drawn around the object and determines the object's height and width. Within the software, a minimum pixel search size is used to remove excess noise that is occasionally included within the HSV thresholding step of the workflow.

### 2.4 Accuracy Metrics

Accuracy metrics, such as precision, recall, the F1 score, and the mean average precision (mAP) have been calculated. Precision is the proportion of correctly identified compared to all actual positive objects (e.g., correctly identified flowers for all flowers in an image). Recall refers to the proportion of correctly identified compared to all identified objects (e.g., correctly identified flowers for all identified objects). F1 scores highlight the balance of precision and recall. When a high F1 score is achieved, it signifies that the detection method performs well across both metrics. The overall detection performance was measured using the mAP score, which is determined by calculating precision at multiple recall levels (totalling 11) and then averaging these precision values over all classes of detected objects. Higher scores indicate better overall model detection performance. The equations used for each measurement can be seen below in Equations (1–5) in Table 2.

**TABLE 2:** Key metrics for model evaluation, including precision, recall, F1 score, average precision, mean average precision, distance to object, latitude and longitude with corresponding mathematical formulas.

Equation	Formula
Precision (1)	$\frac{\text{True Positives}}{\text{True Positives}+\text{False Positives}}$
Recall (2)	$\frac{\text{True Positives}}{\text{True Positives}+\text{False Negatives}}$
F1 Score (3)	$2*\frac{\left(\text{Precision}*\text{Recall}\right)}{\left(\text{Precision}+\text{Recall}\right)}$
Average precision (4)	$\int_{0}^{1}p\left(r\right)\mathrm{dr}$ where *p*(*r*) is the precision as a function of recall.
Mean average precision (5)	$\frac{1}{N}\sum_{i=1}^{N}{\mathrm{AP}}_{i}$ where *N* is the number of classes, and AP_i_ is the average precision for class *i*.
Distance to object (mm) (6)	$\frac{f\left(\mathrm{mm}\right)\times \text{real height}\;\left(\mathrm{mm}\right)\times \text{image height}\;\left(\text{pixels}\right)}{\text{object height}\;\left(\text{pixels}\right)\times \text{sensor height}\;\left(\mathrm{mm}\right)}$
Latitude (7)	*asin*(*sin*(*latcb*)*cos(*hyp*/*r*) + *cos*(*latcb*)*sin(*hyp*/*r*)*cos(*b*))
Longitude (8)	$\text{loncb}+\text{atan}2\left(\sin\left(\mathrm{lb}\right)*\sin\left(\frac{\mathrm{hyp}}{r}\right)*\cos\left(\text{latcb}\right),\cos\left(\frac{d}{R}\right)-\sin\left(\text{latcb}\right)*\sin\left(\text{Latitude}\right)\right)$

### 2.5 Representation of Flowers in a GIS Format

**FIGURE 5:**
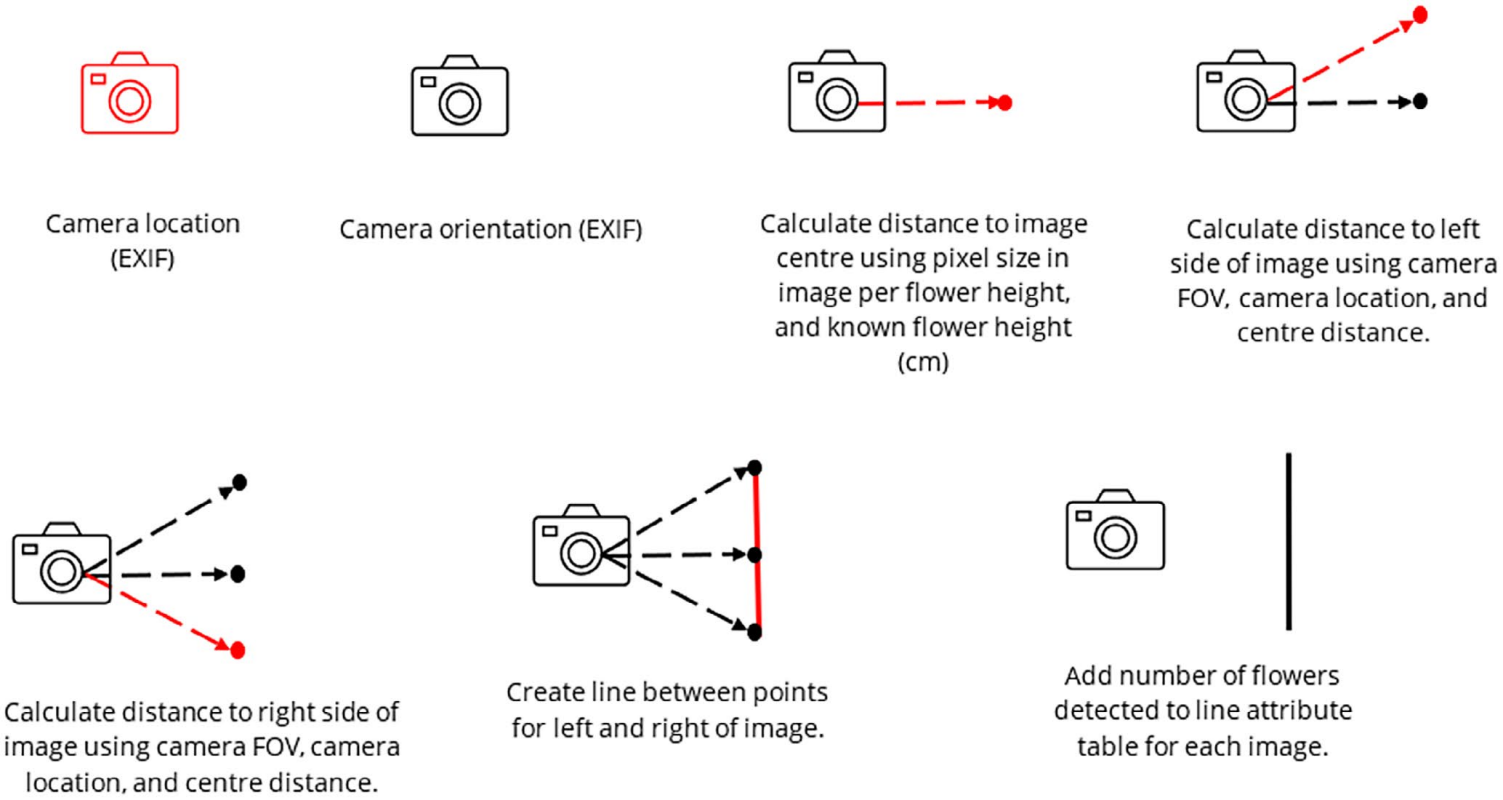
Workflow process to determine the distance between the UAV and the *Impatiens glandulifera* flowers from an image.

Given the images used from the UAV are oblique (i.e., side facing), visualizing the number of classified *Impatiens glandulifera* flowers is not as straightforward as conventional nadir (i.e., top down) imagery. Though methods in the literature do exist for matching oblique features within a georeferenced landscape, they mostly require the use of a DEM and creating tie points using landmark features to georeference the images (Verhoeven et al. 2012; Stockdale et al. 2015; Verykokou and Ioannidis 2016). Given that the EXIF data of the UAV‐derived imagery contains detailed information about the camera setup for each image, this dataset was selected for analysis. Figure 5 provides an overview of the developed workflow process required to determine the *Impatiens glandulifera* flower distance from an image.

Firstly, the position of the camera for each image is attained from the UAV GNSS (Global Navigation Satellite System) coordinates from the image EXIF data, along with the orientation of each image from the camera location. The Phantom 4 Pro has been shown to achieve 2.00 cm relative vertical accuracy and 1.20 cm relative horizontal accuracy when flown at 100 ft. (33 m) (Mulakala 2019). The distance from the camera location to the object was then calculated using Equation (6). The focal length (*f*) and sensor height for the DJI Phantom 4 Advanced camera used are both specified at 8.8 mm. The real height (mm) of the flowers was determined through the measurement of 51 *Impatiens glandulifera* flowers along the River Elwy in North Wales (Eastings: 303458.58, Northings: 371039.85, British National Grid—EPSG:27700) on October 25, 2023. Individual flowers were measured top to bottom using a ruler at random along a walked transect of the riverbank. The mean of 43.2 mm ± 0.7 mm (mean ± SD) was used in Equation (6) in Table 2. Object height in pixels was determined through measuring the height of bounding boxes for all classified flowers per individual image. This therefore resulted in an estimated distance‐to‐object calculation for each image.

Following this, the distances of the left and right side of the image were calculated to provide an image footprint. The specified field of view (FOV) for the camera (73.7°) was halved and subtracted for the image left side and added for the image right side from the initial bearing of the image center (UAV gimbal yaw). These bearings were subsequently converted into radians and used in Equations (7) and (8) in Table 2 to determine the latitude and longitude.

In Equation (7) *latcb* and *loncb* refer to the latitude/longitude bearings for the image center, *hyp* refers to the hypotenuse length, and *r* the radius of the earth (6378.1 km). The variable *b* refers to the bearings for either the left or right side of the image (the equations must be run for each side). *Latitude*, as specified in Equation (8) is the value derived from Equation (7). A line is then created using the latitude and longitude of each image side and saved as a vector file (e.g., GeoJson). The attribute of flowers per meter is then calculated and appended to the vector file by dividing the field of view length by the number of flowers detected via SATT.

## 3 Results

### 3.1 Accuracy Assessment

**FIGURE 6:**
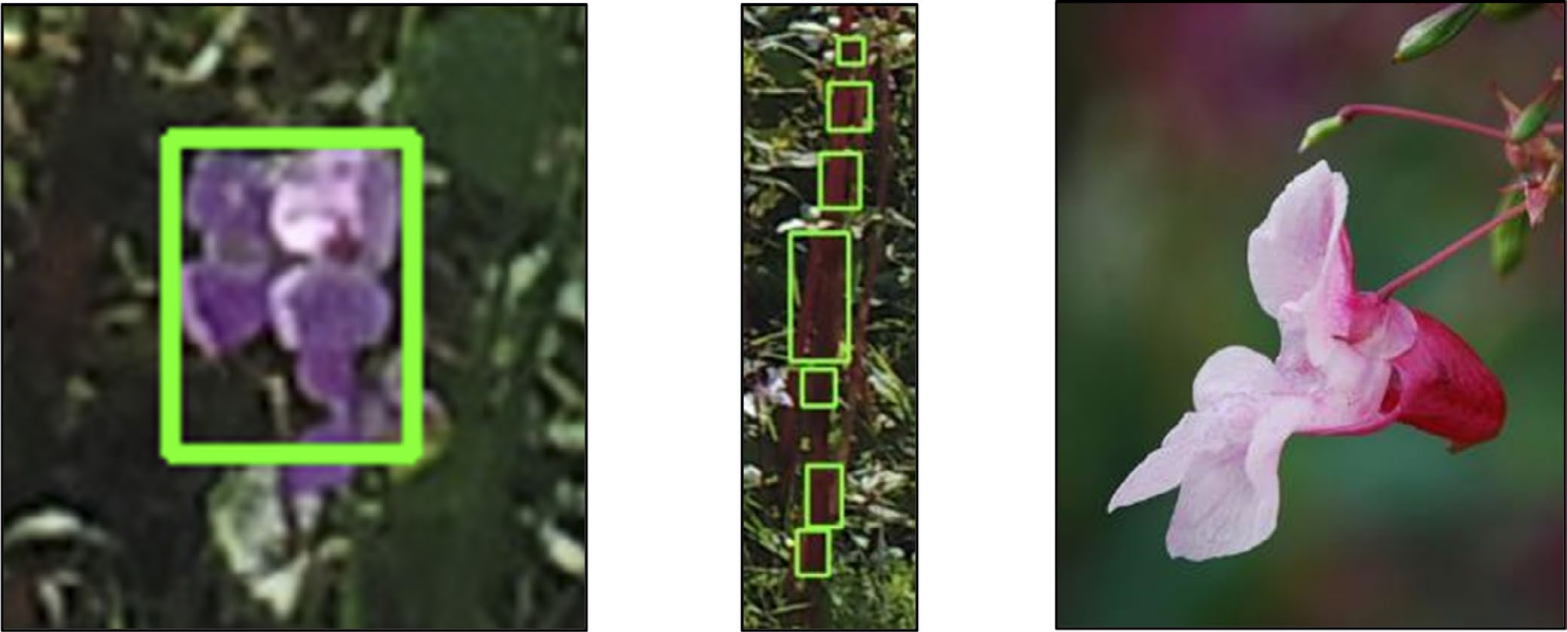
Examples of flower bunching (left image), misclassified purple *Impatiens glandulifera* Stems (central image), and bell‐shaped flowers (right image), sourced from Pixabay, free for use imagery by Kornelia‐Laubach.

**FIGURE 7:**
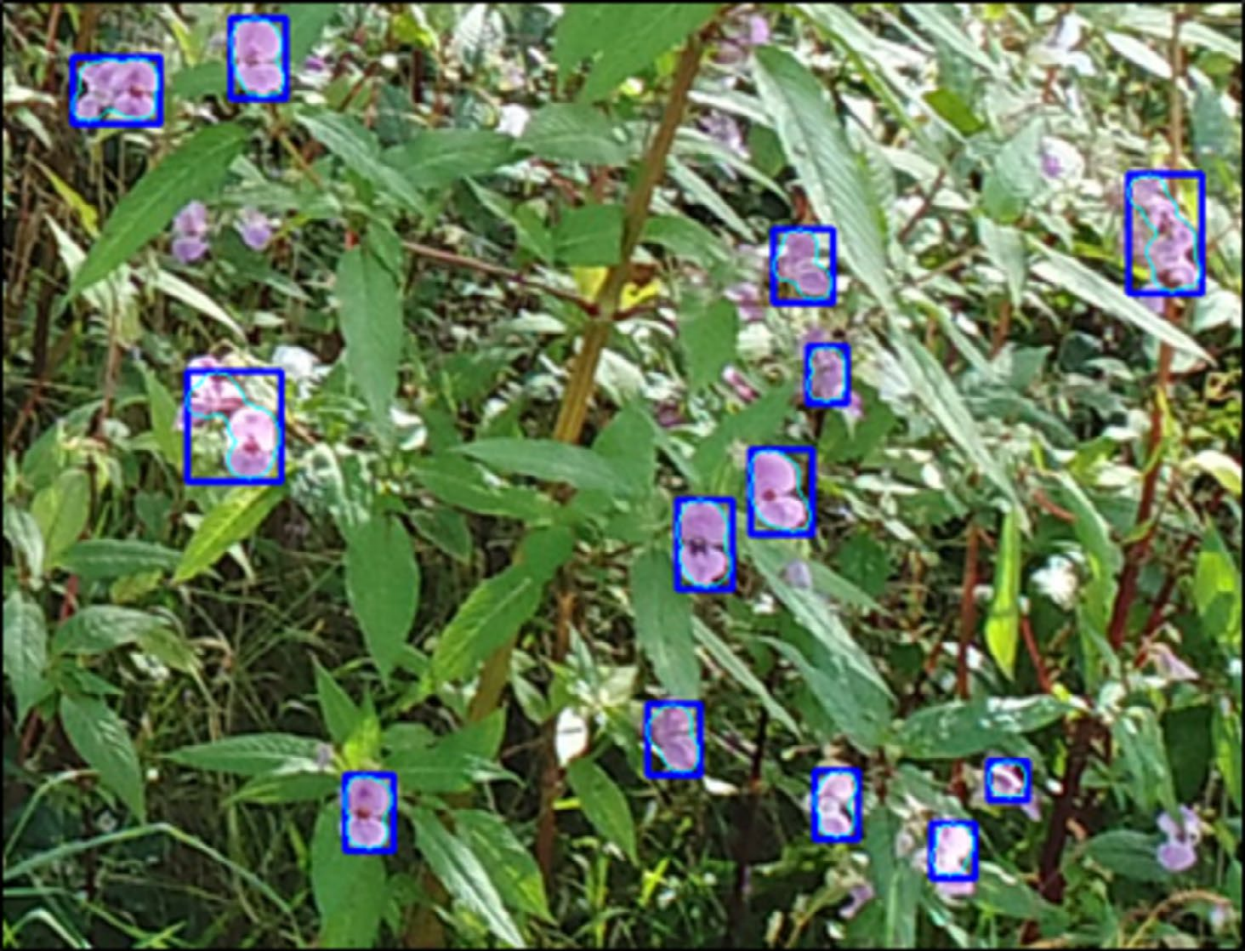
A sample image of *Impatiens glandulifera* flowers that has been annotated using the semi‐automatic thresholding tool. Bounding boxes (blue) and object boundaries (green) have been generated as a result.

To define the accuracy of the SATT workflow, precision, recall, the F1 score, and mAP accuracy measures have been undertaken. Throughout the analysis, flowers that are bunched, overlapping, and boundary‐sharing within the images can often be mistaken for a single flower. Furthermore, as *Impatiens glandulifera* stems share a similar spectral threshold with their flowers, instances arise where the thresholding workflow erroneously classifies a stem as a flower when exposed to direct sunlight. This misclassification occurs because the increased light intensity alters the HSV values of the stems, making them appear similar to the flowers in color. Consequently, the object detection system struggles to distinguish between the two, leading to inaccuracies in the detection process. The algorithm used is further limited by the object detection technique as it searches for ellipsoids, although this can be easily modified to suit other species. The ellipsoid is the shape of the *Impatiens glandulifera* flower from a front‐facing position. However, from the side, the flowers are more bell‐shaped and therefore less likely to be detected. Flowers in the background of the image were more obscured and less definable, and as a result, less likely to be detected, as seen in Figure 6. A total of 312 (99, 108, 105) images from three UAV surveys were analyzed for validation via manual labeling; an example output is shown in Figure 7. Additionally, images captured by the UAV of both sides of the riverbank were excluded from the detection software. This decision was made because the objects in these images were so indistinguishable and unrecognizable to the human eye that any attempt at detection would have been futile. The lack of discernible features and clear differentiation between objects resulted in an image set that could not be reliably processed or analyzed. A random subsample of 10 images from each image set was used to fine‐tune the Hue, Saturation, and Value (HSV) color space threshold parameters within the Advance panel of the GUI. Tuned threshold values for all subsample images were recorded, and the mean of these was used to run the analysis for each image set; these values can be seen in Table 3. In some instances, the pixel size range was also modified to include flowers located further within the background of the image. In each tuning case, the user aimed to extract as many flowers as possible without any misclassification of other detected objects, for example, plant stems. Each image set had differing lighting conditions and viewing angles. Image Set 1 featured optimal lighting conditions and larger, more distinct monoculture stands of *Impatiens glandulifera* . In contrast, Image Sets 2 and 3 lacked these favorable conditions and exhibited smaller, less defined plant stands. Each image was examined by a user to count identifiable flowers. Flowers that were obscured or challenging to identify by the user were not included in the count. These were then compared to the amount that were correctly classified as flowers and incorrectly classified (e.g., stems) by the threshold workflow.

**TABLE 3:** Mean upper and lower HSV thresholds and minimum object size (pixels) used for the processing of each image set.

		Image Set 1	Image Set 2	Image Set 3
Lower threshold	H	117	115	118
	S	9	29	23
	V	0	0	0
Upper threshold	H	230	221	225
	S	200	144	200
	V	0	0	0
Minimum object size (*X*, *Y*)		15, 15	6, 7	10, 12

**FIGURE 8:**
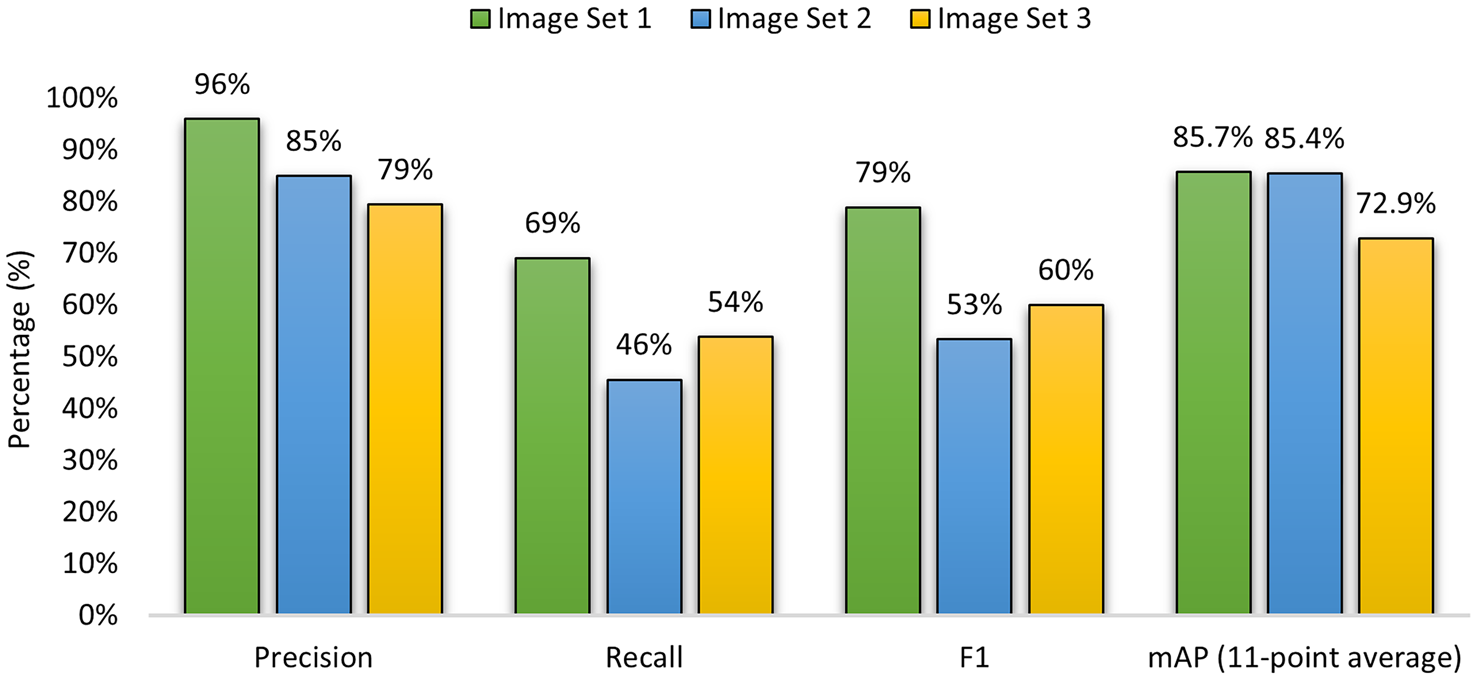
Accuracy metrics for each image set, including Precision, Recall, F1 score, and the mean Average Precision Scores using the 11‐point average.

**FIGURE 9:**
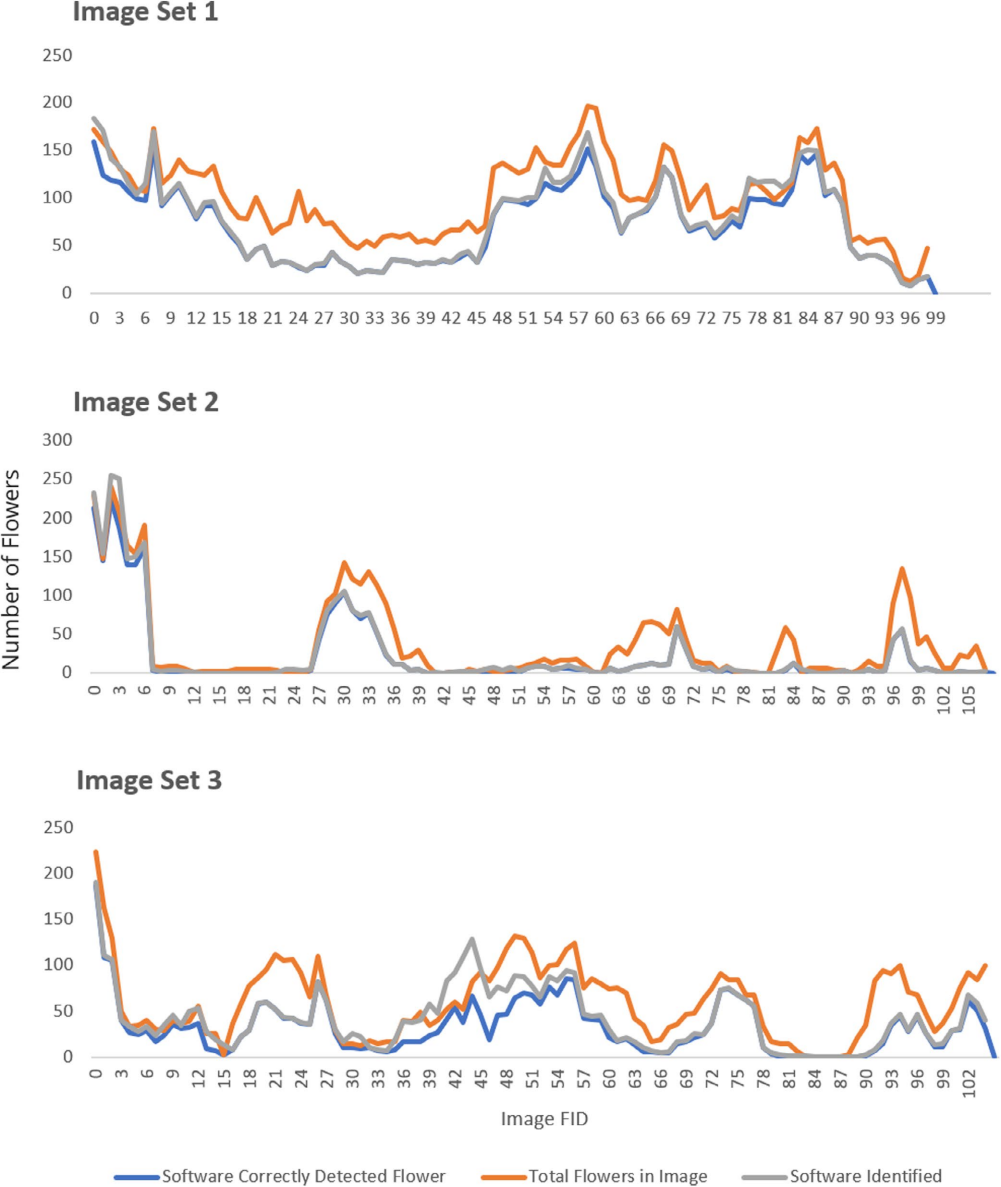
A comparison between the software correctly detected flowers, the total number of flowers observed, and software identified flowers in each image set.

In summary, the accuracy metrics show that the software has been able to obtain a high precision score (> 79%) but recall could be improved (46%–69%). The F1 scores range from 53% to 79% and highlight the impact of lower recall scores in comparison to the precision scores. The mAP alongside the Precision, Recall, and F1 metrics are plotted in Figure 8. The model obtained an accuracy rating of 85.7%, 85.4%, and 72.9% for Image Sets 1, 2, and 3, respectively. This performance allows for the possible application of the software for land management uses and invasive species mitigation, as the high precision of the software is ideal for users who value minimizing false positive readings. Figure 9 highlights the relationship between the software detected flowers and the actual flowers within the image.

### 3.2 Visualization

**FIGURE 10:**
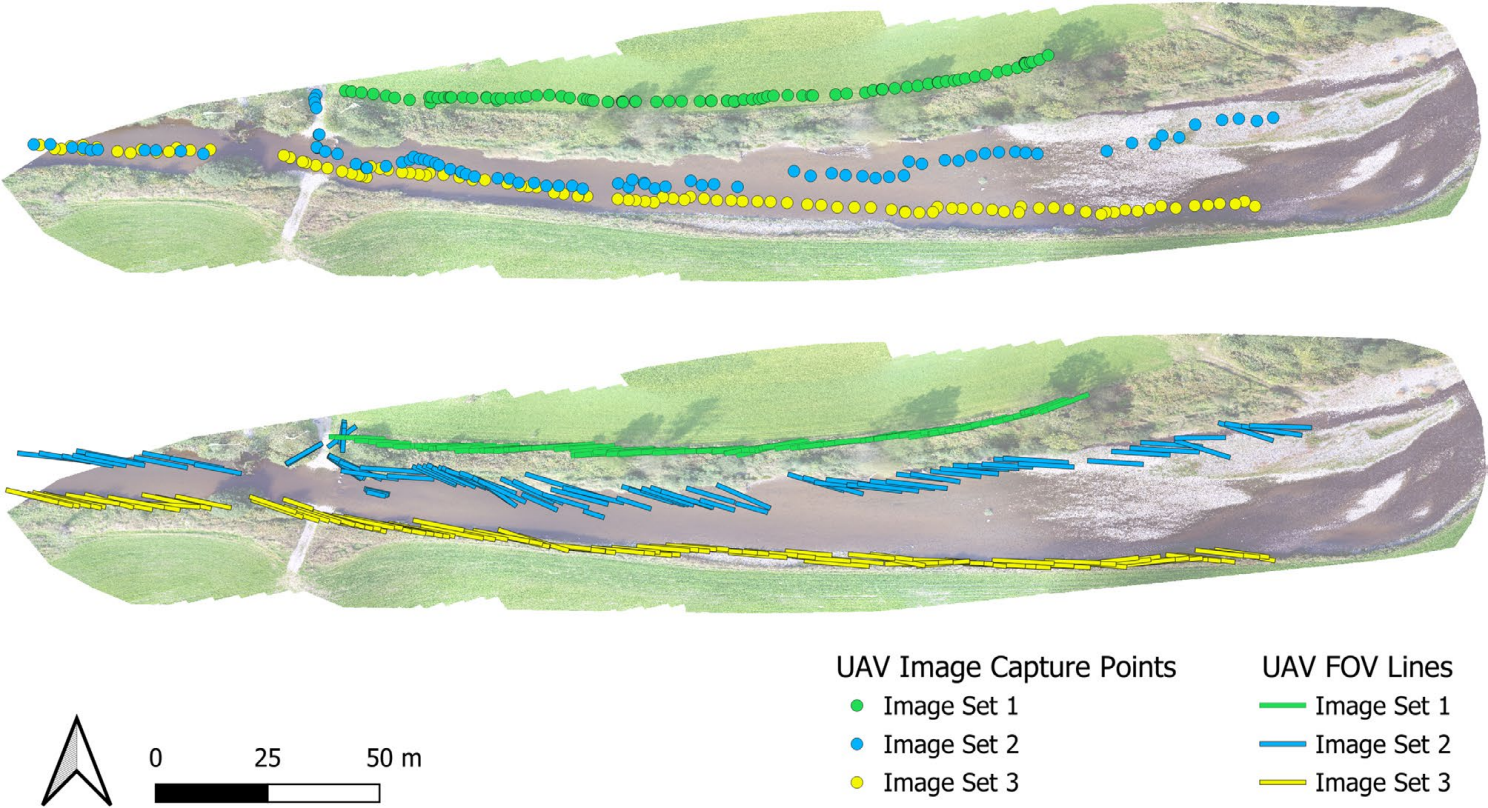
Visualization example of the UAV survey image capture points and field of view (FOV).

**FIGURE 11:**
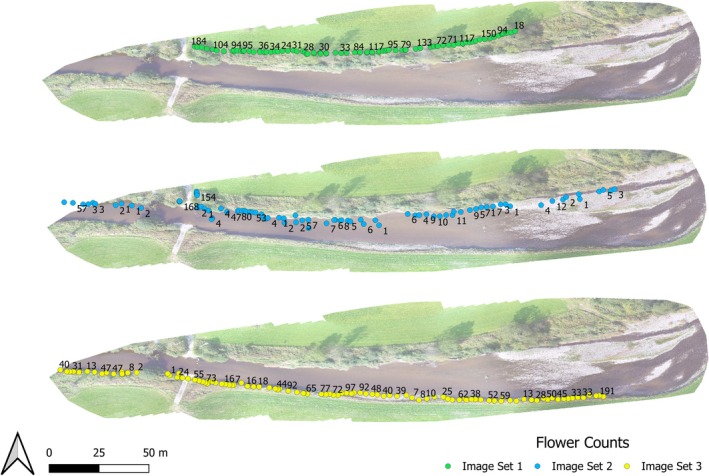
Visualization example of the detected objects/flowers of *impatiens glandulifera* within each image. Numbers represent the number of detected flowers within each image. Images are positioned based on the mean distance of all detected objects.

**FIGURE 12:**
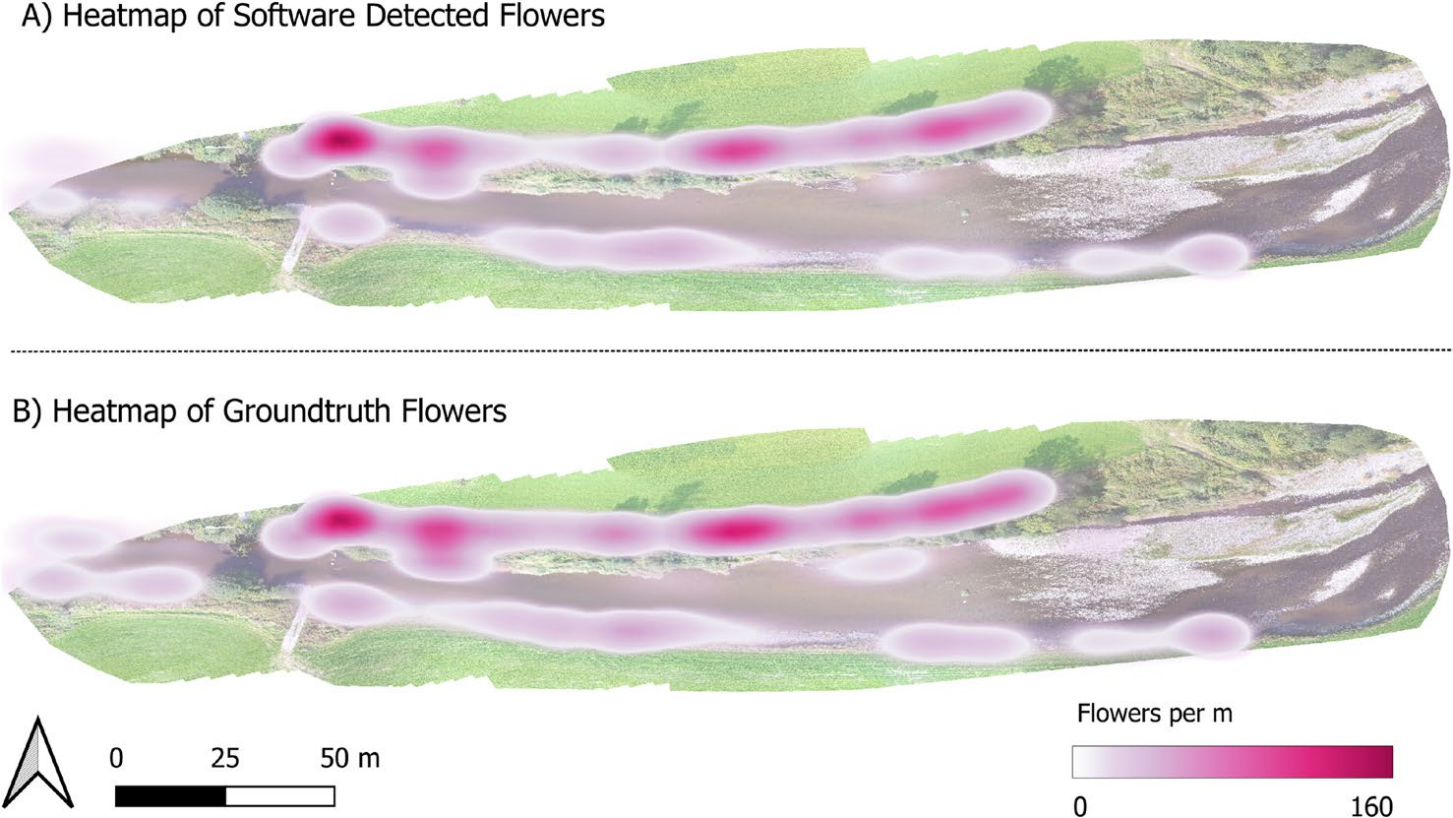
Heatmaps of *Impatiens glandulifera* using the flower per meter as a weighted variable for the semi‐automatic thresholding tool (SATT) detected flowers (A) and groundtruth flowers (B).

The UAV center points, Field of View (FOV) and object points can all be visualized as vector datasets for use in GIS. The center points represent the UAV position during image capture intervals, as seen in Figure 10. The FOV lines demonstrate the extent of the image on the ground and are calculated from determining the left and right‐most object distance within each image. The flower count variable calculated as part of the main semi‐automatic thresholding workflow provides an estimation of detected objects within each image. This can then be displayed via labels, as seen in Figure 11, or as a heat map with the detected objects variable used as a weight, as seen in Figure 12. In Figure 11, multiple location points are within the river due to the reflection of the flowers upon the water surface.

## 4 Discussion

The SATT's high mAP scores of 72%–86% across different image sets underscore its robustness and accuracy, corroborating findings from similar studies using semi‐automatic methods for invasive species detection (Martínez‐Sánchez et al. 2019; Rotger et al. 2019). For example, one study focused on mapping the invasive alien species *Acacia dealbata* in NW Spain, combining UAV surveys with Sentinel‐2 imagery and automating part of the process through scripting for querying, downloading, and mosaicking satellite images (Martínez‐Sánchez et al. 2019). Another study utilized the Automatic Photo Identification Suite (APHIS) to identify snakes from photographs by analyzing natural markings and scale patterns on their heads (Rotger et al. 2019). The suggestion that higher detection accuracies could be achieved by splitting image sets into smaller sub‐groups for fine‐tuning highlights an area for future research and algorithm optimization.

The high recall error observed in this study aligns with previous findings that UAV altitude and object size significantly impact detection accuracy (Mittal et al. 2020). As UAVs move further away from target objects, the resolution decreases, making smaller objects harder to detect (Redmon 2016; Du et al. 2018). Therefore, when a minimum object size is set by the user, any flowers that are below this size will not be detected. Furthermore, during the fine‐tuning phase, the user focused on under‐classifying flower objects so that false positive detections were avoided as much as possible, and specifically on classifying the purple color of the flowers rather than the white colored flowers. This approach was implemented to maintain the precision and reliability of the detections, as the presence of false positives could undermine the overall effectiveness and credibility of the system (Ajadi et al. 2016).

It's important to note that balsam flowers can be naturally white, and lightly pink flowers, when illuminated by bright sunlight, can also appear white (Burdziakowski and Bobkowska 2021; Ramachandran and Sangaiah 2021). Consequently, these pink flowers appearing white under bright conditions were not detected, highlighting a potential limitation in the detection process and suggesting a need for enhanced colour calibration methods or the integration of additional spectral bands to improve detection under varying light conditions (Bradley 2014; Burdziakowski and Bobkowska 2021). The detection of flower reflections on water surfaces highlights an area for further refinement, potentially through the application of advanced image filtering techniques to distinguish true objects from reflective artefacts (Busch et al. 2013; Yu et al. 2020).

The SATT processes individual images, yielding rapid image analysis in the context of invasive species detection. This capability enables potential real‐time processing, reduces the need for extensive post‐processing, and streamlines fieldwork by eliminating the requirement for ground control points. Utilizing the EXIF data from these single images, the locations of the detected flowers have been mapped. Therefore, this approach offers a practical solution for location monitoring, even within hazardous and challenging terrain. Additionally, it supports change detection through routine surveys and augments the available data for informed, data‐driven decision making in land management.

Figures 9 and 12 both reveal consistent trends between the SATT‐detected flowers and the ground truth data. Notably, Figure 12 illustrates a robust correlation in hotspot zones across the SATT‐detected flowers and the ground truth data. Consequently, this finding holds promise for hotspot identification, potentially enhancing early detection and management strategies in these areas. Furthermore, it may facilitate more effective resource allocation and conservation planning.

In comparison to Convolutional Neural Networks (CNNs), the SATT's intuitive design and minimal training data requirements offer a user‐friendly alternative, facilitating broader adoption amongst nonexperts. The simplicity and possibility of running the program also enable it to be run on a single‐board computer for real‐time detection and processing of flowers. Despite this, the tool does record annotations in a format usable by CNNs, highlighting the potential for future methodological evolution. If a wide range of datasets is obtained, the application of robust CNN models could improve detection accuracy and generalization across varying conditions, including changes in lighting or object sizes. However, the computational demands and extensive training data required for CNNs necessitate further investigation into their feasibility for rapid assessment or real‐time applications. By accommodating image conditions to ensure consistency, this study bridges the gap between accessible, semi‐autonomous detection tools and more sophisticated machine learning methodologies.

### 4.1 Limitations and Future Work

The SATT should be further tested with imagery of varying lighting, contrasts, and with differing species of similar flowering time to understand what limits to the flower detection process the software may possess. Existing limitations in this study example include the misclassification of bunched flowers as a single flower, the misclassification of *Impatiens glandulifera* stems as flowers, the white *Impatiens glandulifera* flowers omitted from detection, and bell‐shaped flowers omitted due to the ellipsoid kernel shape used.

Furthermore, the datasets only incorporate flowers from a single survey area; a larger dataset should be obtained from multiple survey areas to account for environmental influence on image capture and *Impatiens glandulifera* density, flower size, and colouration. Moreover, the study site we assessed did not incorporate multiple species with similar flower characteristics, of which the SATT tool may struggle to delineate between these. For example, in this study if there was a higher occurrence of rosebay willowherb ( *chamaenerion angustifolium* ), there may have been an increase of false positives identified by the SATT due to the similar coloring and shape of flowers to *Impatiens glandulifera* .

The SATT software is still in an early iteration, but future improvements of the software to improve usability could include automatic image set splitting based on the mean image brightness or variance amongst each image for easier fine‐tuning of the image subsets. Additionally, the SATT software could run multiple thresholding workflows to extract higher quantities of objects based on multiple user input thresholds. This might be useful in cases where generic HSV values of the desired object encapsulate small HSV ranges for other undesirable objects.

## 5 Conclusions

The SATT has a high mAP score of 72%–86%, meaning a low chance of false positive detections. More importantly, the software detection rate is on trend with total objects per image and enables a reliable hotspot/relative difference to be determined (and in essence a rank of worst‐to‐least affected areas to be calculated). Therefore, this enables appropriate targeting of land management techniques to control invasive species in harder‐to‐access zones along riparian corridors.

The ease of use for the tool, requiring no expert knowledge of coding, scientific expertise, or data manipulation, makes the SATT accessible to everyone. Users can effortlessly fine‐tune the tool parameters through the SATT advanced window, adjusting threshold values for hue, saturation, and lightness via widget sliders. This user‐friendly interface allows for efficient iterations and immediate visual feedback within the software graphics widget. The SATT then automatically transforms detected objects into GIS vector layers, providing ready‐to‐use visual data layers for further analysis, such as hotspot mapping.

The SATT can be adapted to other objects or invasive species by fine‐tuning the tool parameters with the SATT advanced window, although the flowers/objects must be sufficiently different from surrounding objects either by shape, size, or colors but ideally all three. This adaptability extends the utility of the SATT beyond a single application.

Further research should seek to understand if the SATT would function effectively when similar objects sharing the same colorations and size characteristics are located within the same image. Different detection methods should also be incorporated to enable applications to different environmental situations.

In summary, the SATT is a rapid assessment tool that alleviates the need for manual image review from UAV surveys, facilitating user operations without replacing human analysis or functioning as a standalone system.

## Author Contributions

**Jack Cook:** conceptualization (equal), data curation (equal), formal analysis (lead), investigation (lead), methodology (equal), resources (lead), software (lead), validation (lead), visualization (lead), writing – original draft (lead), writing – review and editing (equal). **Benjamin P. Roberts:** conceptualization (equal), data curation (equal), formal analysis (equal), investigation (equal), methodology (equal), supervision (equal), validation (equal), visualization (supporting), writing – review and editing (equal). **Frédéric Labrosse:** methodology (equal), supervision (equal), writing – review and editing (equal). **Neal Snooke:** methodology (equal), supervision (equal), writing – review and editing (equal).

## Conflicts of Interest

The authors declare no conflicts of interest.

## Data Availability

The raw data and code used in this study are available in the public repository on GitHub. The dataset includes images of *Impatiens glandulifera* captured using a Phantom 4 multispectral UAV along the River Elwy in Wales. The code repository contains scripts for software compiling and data analysis. Researchers interested in reproducing or building upon the findings can access the data and code through this link: https://github.com/jrc15/SATT.
